# Usefulness of lactate to albumin ratio for predicting in-hospital mortality in atrial fibrillation patients admitted to the intensive care unit: a retrospective analysis from MIMIC-IV database

**DOI:** 10.1186/s12871-024-02470-4

**Published:** 2024-03-21

**Authors:** Ting Huang, Sen Lin

**Affiliations:** 1 Department of Cardiology, The Central Hospital of Wuhan, Tongji Medical College, Huazhong University of Science and Technology, Wuhan, 430014 Hubei China; 2 Key Laboratory for Molecular Diagnosis of Hubei Province, The Central Hospital of Wuhan, Tongji Medical College, Huazhong University of Science and Technology, Wuhan, 430014 China; 3 Department of Cardiology, Zhongnan Hospital of Wuhan University, Wuhan, 430071 Hubei China

**Keywords:** Atrial fibrillation, Intensive care unit, Predict, In-hospital mortality

## Abstract

**Background:**

High lactate to albumin ratio (LAR) has been reported to be associated to with poor prognosis in patients admitted to the intensive care unit (ICU). However, its role in predicting in-hospital mortality in AF patients admitted to ICU has not been explored.

**Methods:**

The Medical Information Mart for Intensive Care-IV (MIMIC-IV) database was used to retrieve information on patients who had been diagnosed with AF. X-tile software was utilized to determine the optimal cut-off LAR. Area under the receiver operating characteristic curves (AUC), calibration plots, and decision curve analysis (DCA) were conducted to assess the prediction performance of LAR for in-hospital mortality.

**Results:**

Finally, 8,287 AF patients were included and 1,543 death (18.6%) occurred. The optimal cut-off value of LAR is 0.5. Patients in lower LAR (< 0.5) group showed a better in-hospital survival compared to patients in higher LAR (≥ 0.5) group (HR: 2.67, 95%CI:2.39–2.97, *P* < 0.001). A nomogram for in-hospital mortality in patients with AF was constructed based on multivariate Cox analysis including age, CCI, β blockers usage, APSIII, hemoglobin and LAR. This nomogram exhibited excellent discrimination and calibration abilities in predicting in-hospital mortality for critically ill AF patients.

**Conclusion:**

LAR, as a readily available biomarker, can predict in-hospital mortality in AF patients admitted to the ICU. The nomogram that combined LAR with other relevant variables performed exceptionally well in terms of predicting in-hospital mortality.

**Supplementary Information:**

The online version contains supplementary material available at 10.1186/s12871-024-02470-4.

## Introduction

Atrial fibrillation (AF) is one of the most common arrhythmias in intensive care unit (ICU) patients, impacting up to 25% of patients hospitalized to ICU for non-cardiac surgery [1, 2]. AF is estimated to account for 47.4–61% of all arrhythmias and 52% of atrial arrhythmias in critically ill patients [3, 4]. AF is associated with worse outcomes, AF not only increases the risk of stroke in ICU patients, but also significantly increases their risk of in-hospital mortality. A study based on the nationwide German inpatient sample reported that in-hospital death rate was significantly higher in surgical patients with AF (6.3%) than those without AF (1.1%) [5]. Another study indicated that AF in ICU, whether new-onset or recurrent, is independently associated with increased in-hospital mortality [6]. FROG-ICU study revealed that new-onset AF showed 1.6-time risk of in-hospital death compared to no AF [7]. Hence, it’s important to develop a simple and easily obtainable predictor to screen those in high-risk in-hospital mortality AF patients admitted to ICU.

Recently, many researchers have proposed lactate to albumin ratio (LAR) as an important predictive factor for predicting the prognosis of ICU patients in different diseases settings [8–10]. Elevated serum lactate levels could reflect poor tissue perfusion and disease severity due to tissue hypoxia and anaerobic metabolism [11]. Albumin is a negative acute phase protein, is mainly synthesized by the liver and has multiple important physiological functions, which reflects the nutritional status, inflammation, and chronic disease status [12]. LAR can provide a variable that includes comprehensive information about the patient’s nutrition and other physiological changes including nutritional status, inflammation, etc. LAR reported to have predictive value for predicting 30-day mortality in patients with acute myocardial infarction [13]. Moreover, LAR could serve as a predictor of short and long-term mortality in critically ill patients with heart failure [14]. However, the predictive role of LAR in AF patients admitted to ICU remains largely unknown.

Here, we aimed to evaluate the in-hospital mortality predictive role of LAR in AF admitted to ICU. We hypothesized that the LAR could predict in-hospital mortality in critically ill patients with AF.

## Methods

### Data source

All information was taken from the Medical Information Mart for Intensive Care-IV (MIMIC-IV version 1.0) database, which is an upgraded MIMIC-III version that has previously received institutional review board approval [15]. Created by the Massachusetts Institute of Technology (MIT) computational physiology lab and approved by the institutional review boards of both MIT and Beth Israel Deaconess Medical Center (BIDMC), the MIMIC-IV currently contains comprehensive and high-quality data of patients admitted to intensive care units (ICUs) at the Beth Israel Deaconess Medical Center (BIDMC) between 2008 and 2019. The MIMIC-IV database provides demographics, vital signs, laboratory markers, and pharmaceutical information for more over 50,000 critically ill patients at BIDMC between 2008 and 2019. It also incorporates desensitization data. Due to the data used in this study were extracted from public databases, it was exempt from the requirement for informed consent from patients and approval of the ethics review committee. After finishing the web-based training courses and the Protecting Human Research Participants examination, one author (Ting Huang) was approved to extract data from the MIMIC-IV (Record ID: 37,474,354).

### Cohort selection

**Fig. 1:**
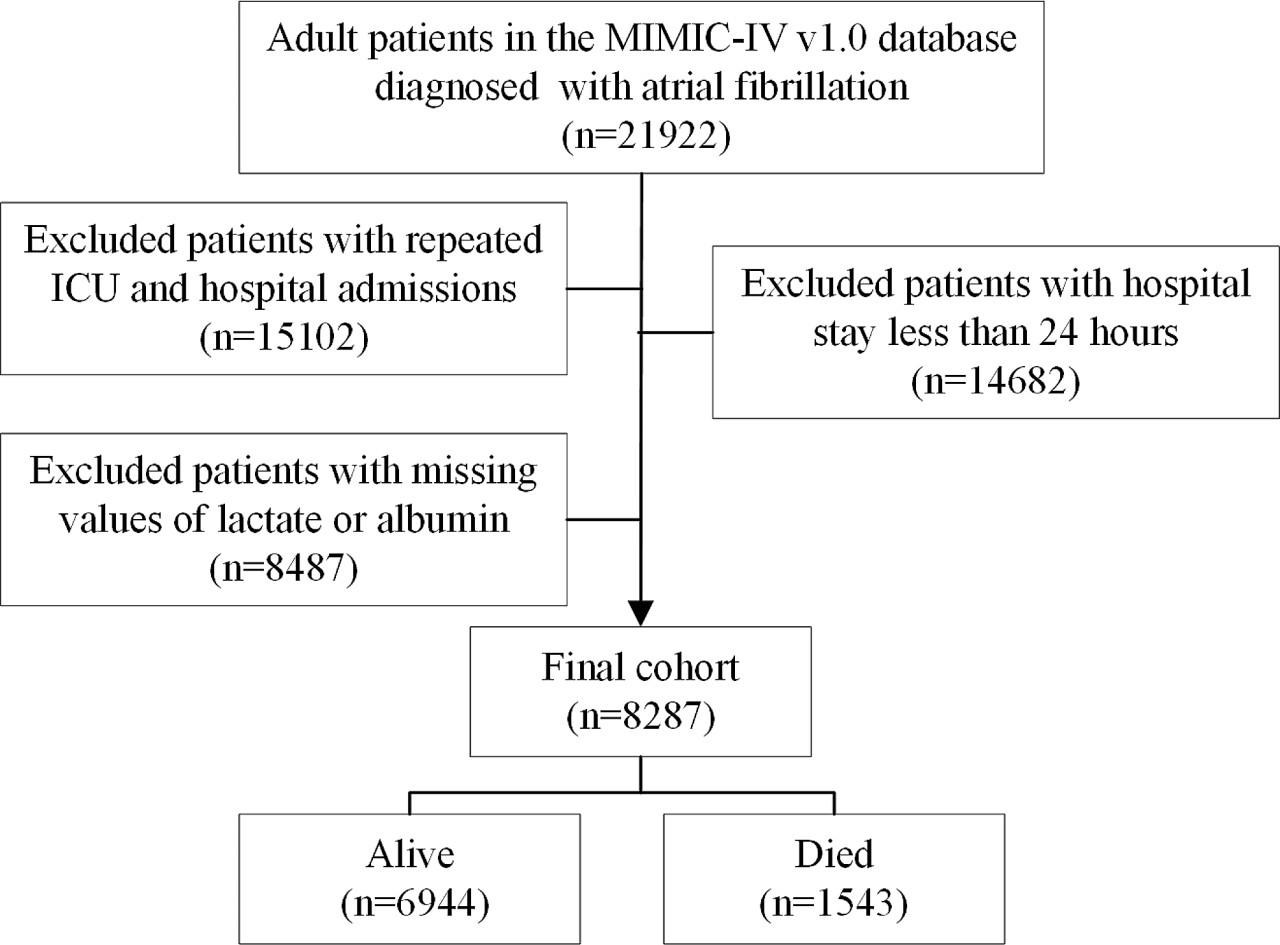
The flow chart of this study

AF was diagnosed according to ICD-9 (42,731), and ICD-10 (I480, I481, I482, and I4891). The exclusion criteria were as followings: (1) hospital stay less than 24-hours; (2) missing data of lactate or albumin; (3) patients with repeated ICU admissions. Finally, 8,287 AF patients were included (Fig. 1).

### Date collection and outcomes

All variables were collected at the 24-h after the admission. Baseline parameters including age, gender, weight, comorbidities, Charlson comorbidity index, drug usage, inventions, Sequential Organ Failure Assessment score (SOFA), Simplified Acute Physiology Score II (SAPSII) and oxford acute severity of illness score (OASIS), acute physiology score III (APSIII), vital signs. The laboratory values were also included. Meanwhile, clinical outcomes included length of ICU stay, length of hospital stay were also included. The primary outcome was in-hospital mortality.

### Statistical analysis

Baseline characteristics are expressed as mean ± standard deviation (SD) and categorical variables are expressed as number (percentage). The baseline characteristics between different groups were compared using analyzed with Student’s t test or chi-square test. The X-tile software was conducted to evaluate the optimal cut-off LAR. Furthermore, the selection of important determinants for in-hospital mortality in patients with AF was performed by the least absolute shrinkage and selection operator (LASSO) regression. After that, a nomogram for the prognosis of critically ill AF patients was created using multivariable logistic analysis. Moreover, univariate and multivariate Cox regression were conducted to determine the nomogram for prediction of in-hospital mortality. Area under the receiver operating characteristic curves (AUC), calibration plots, and decision curve analysis (DCA) were conducted to assess the prediction performance of the predictive nomogram for in-hospital mortality.

## Results

### Patient characteristics

**Fig. 2:**
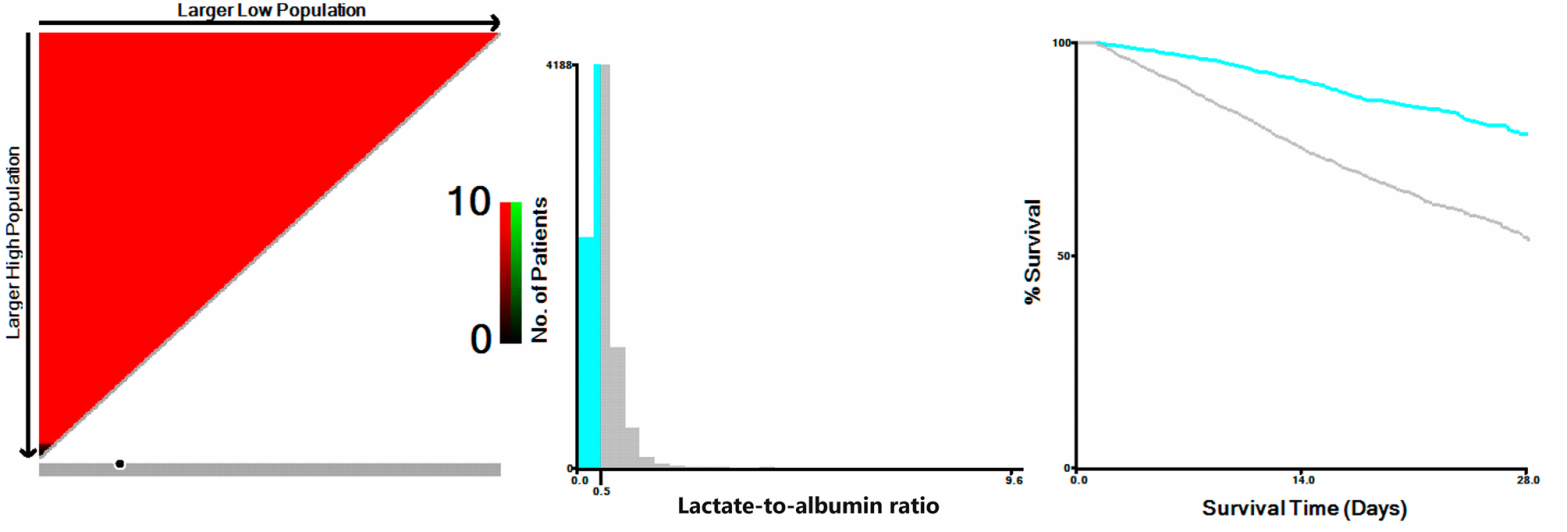
X-tile analyses to obtain the optimal cut-off value of lactate-to-albumin ratio. X-tile plots for patients with critically ill atrial fibrillation are shown on the left panels; the black circles indicate the optimal cut-off values, which are also presented in histograms (middle panels). Kaplan-Meier curves are shown in the right panels

Finally, 8,287 AF patients were included in the present study. 1,543 death (18.6%) occurred (Fig. 1). The optimal cut-off value of LAR is 0.5. Then these patients were divided into two groups according to the optimal cut-off value (Fig. 2). Table 1 exhibited the baseline characteristics.

**Table 1:** The baseline characteristics of all patients

Characteristics	Low LAR (*N* = 4955)	High LAR (*N* = 3532)	*P* value
Age, years old	74.3 ± 11.7	74.8 ± 11.9	0.096
Gender, male, *n* (%)	2901 (58.5)	2074 (58.7)	0.873
Weight, kg	82.6 ± 24.5	82.0 ± 23.5	0.232
Comorbidities, *n* (%)			
Myocardial infarction	1219 (24.6)	875 (24.8)	0.856
Congestive heart failure	2534 (51.1)	1812 (51.3)	0.883
Hypertension	2101 (42.4)	1250 (35.4)	< 0.001
Diabetes	1679 (33.9)	1242 (35.2)	0.221
Chronic kidney disease	1511 (30.5)	1153 (32.6)	0.035
Charlson comorbidity index, points	6.8 ± 2.5	7.3 ± 2.6	< 0.001
Drugs usage, *n* (%)			
βblockers	4265 (86.1)	2913 (82.5)	< 0.001
Statins	2979 (60.1)	1748 (49.5)	< 0.001
Aspirin	3543 (71.5)	2087 (59.1)	< 0.001
Interventions, *n* (%)			
Mechanical ventilation	2861 (57.7)	1846 (52.3)	< 0.001
Vasopressors	2576 (52.0)	1894 (53.6)	0.137
Score system, points			
SOFA	5.8 ± 2.5	7.0 ± 2.8	< 0.001
OASIS	33.4 ± 8.9	36.7 ± 9.8	< 0.001
APSIII	50.7 ± 23.0	62.3 ± 27.3	< 0.001
SAPSII	40.1 ± 12.6	44.8 ± 14.2	< 0.001
Vital signs			
Mean arterial pressure, mmHg	81.3 ± 18.5	80.6 ± 18.9	0.062
Heart rate, bpm	87.3 ± 20.9	93.8 ± 23.4	< 0.001
Laboratory values			
White blood cell, × 10^9^/L	10.7 ± 3.5	12.7 ± 4.7	< 0.001
Hemoglobin, g/dL	11.2 ± 2.3	10.8 ± 2.3	< 0.001
Platelet, × 10^9^/L	211.9 ± 97.1	211.2 ± 98.9	0.776
Albumin, g/dL	3.5 ± 0.6	3.0 ± 0.6	< 0.001
Lactate,	1.2 ± 0.3	2.4 ± 1.3	< 0.001
LAR	0.34 ± 0.09	0.82 ± 0.24	< 0.001
Creatinine, mg/dL	1.1 ± 0.3	2.2 ± 0.8	< 0.001
Bicarbonate,	24.5 ± 4.7	23.1 ± 4.9	< 0.001
Outcomes			
Length of ICU stay	2.6 (1.4, 5.1)	3.2 (1.7, 6.7)	< 0.001
Length of hospital stay	9.9 (6.6, 25.3)	10.9 (6.6, 28.9)	< 0.001
In-hospital death	488 (9.8)	1055 (29.9)	< 0.001

APSIII, acute physiology score III, ICU, intensive care unit, LAR, lactate-to-albumin ratio, OASIS, oxford acute severity of illness score, SAPS II, simplified acute physiology score II, SOFA, sequential organ failure assessment

### LAR was an independent predictor for in-hospital mortality

**Fig. 3:**
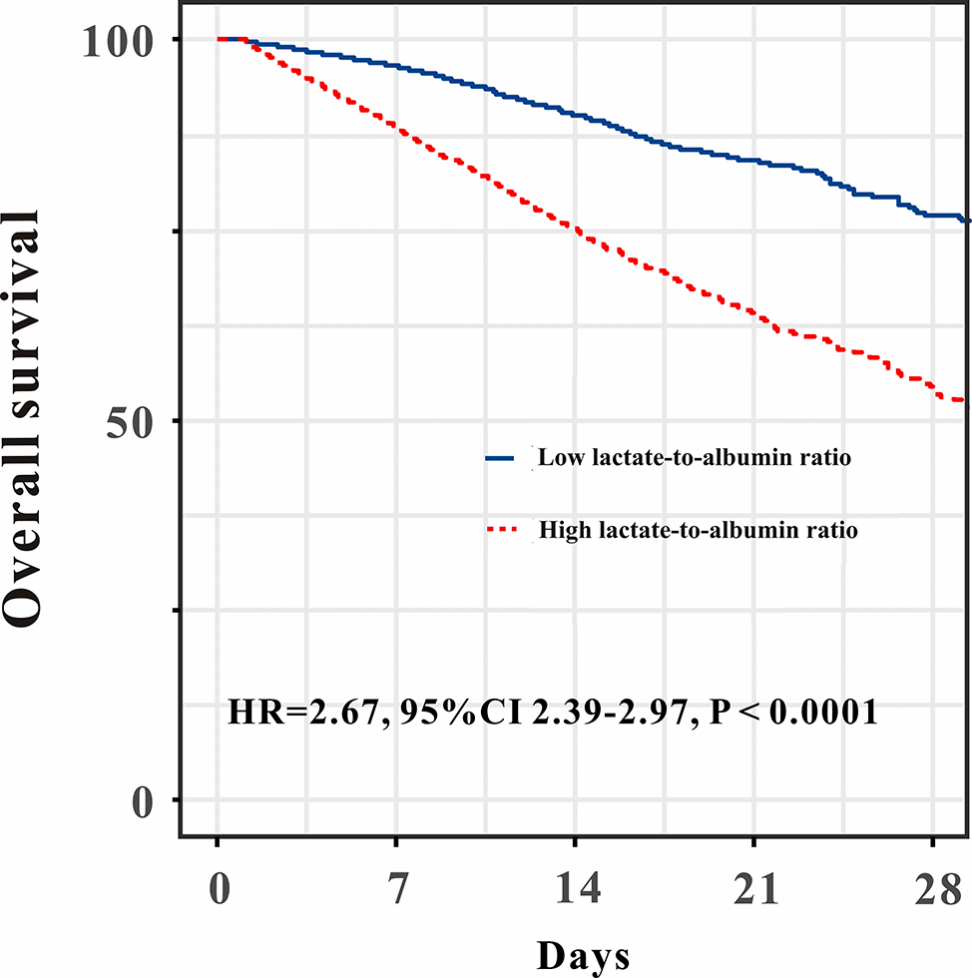
Overall survival of high-lactate-to-albumin ratio and low-lactate-to-albumin ratio groups conducted by Kaplan-Meier curve

Kaplan-Meier analysis indicated that patients in lower LAR (< 0.5) group showed a better in-hospital survival compared to patients in higher LAR (≥ 0.5) group (HR: 2.67, 95%CI:2.39–2.97, *P* < 0.001, Fig. 3). Moreover, the AUC for the prognostic factor LAR was 0.721 (0.707–0.737), exhibited good in-hospital mortality predictive ability (Fig. 6B).

#### Nomogram incorporated with LAR showed excellent discriminative capacity

**Fig. 4:**
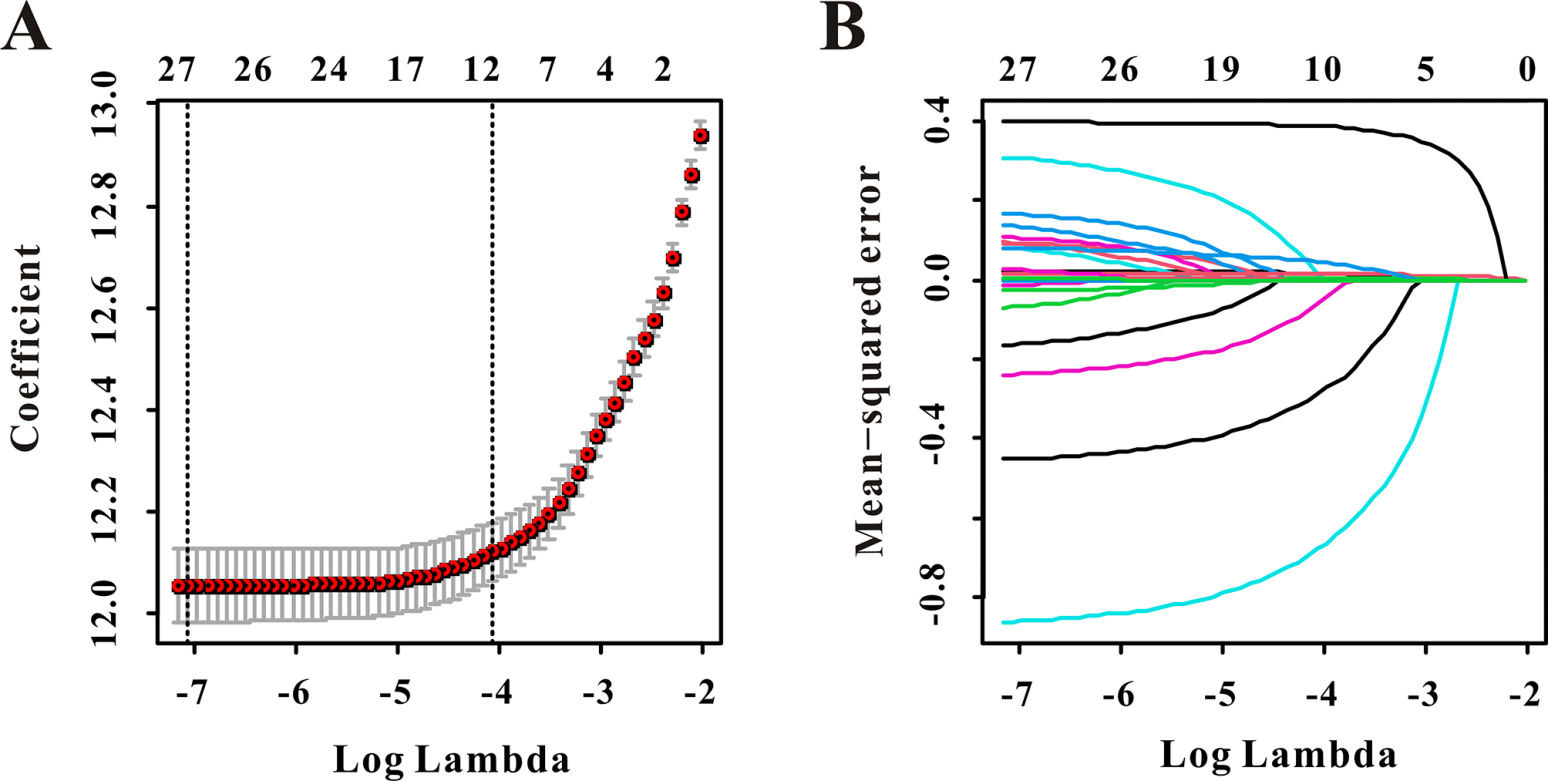
Selection of significant factors associated with in-hospital mortality in critically ill atrial fibrillation patients by LASSO Cox regression model. **(A)** The selection process of the optimum value of the parameter λ in the Lasso regression model by cross-validation method; **(B)** the variation characteristics of the coefficient of variables

**Fig. 5:**
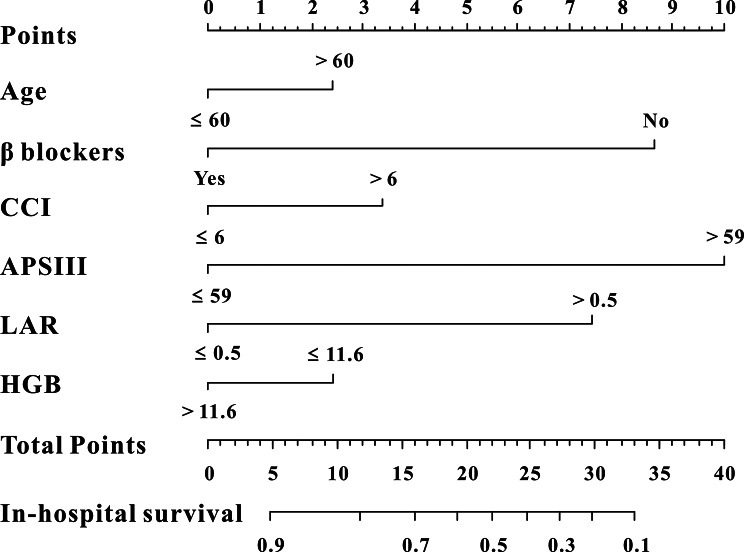
The survival nomogram for predicting in-hospital mortality of critically ill patients with atrial fibrillation. When using it, drawing a vertical line from each variable upward to the points and then recording the corresponding points (i.e., “age > 60 years old” = 0.8 points). The point of each variable was then summed up to obtain a total score that corresponds to a predicted probability of in-hospital mortality at the bottom of the nomogram

Six risk factors were eventually included in this study, as shown by LASSO regression for in-hospital mortality prediction (Fig. 4A-B). Furthermore, a nomogram for in-hospital mortality in patients with AF was constructed based on multivariate Cox analysis (Table 2). Age (HR: 1.03, 95%CI:1.02–1.04, *P* < 0.001), CCI (HR: 1.09, 95%CI:1.06–1.11, *P* < 0.001), β blockers (HR: 0.40, 95%CI:0.35–0.44, *P* < 0.001), APSIII (HR: 1.02, 95%CI:1.01–1.03, *P* < 0.001), Hemoglobin (HR: 0.96, 95%CI:0.94–0.99, *P* = 0.002) and LAR (HR: 1.52, 95%CI:1.44–1.60, *P* < 0.001) were finally included in the nomogram (Fig. 5).

**Table 2:** Logistic regression analysis for the risk factors of in-hospital mortality selected by LASSO regression

	Univariate		Multivariate	
	HR (95% CI)	*P*	HR (95% CI)	*P*
Age	1.03 (1.02–1.04)	< 0.001	1.03 (1.02–1.04)	**< 0.001**
Myocardial infarction	1.18 (1.06–1.32)	0.003	1.11 (0.99–1.25)	0.071
Diabetes	0.95 (0.86–1.06)	0.378		
CCI	1.12 (1.10–1.14)	< 0.001	1.09 (1.06–1.11)	**< 0.001**
β blockers	0.37 (0.33–0.41)	< 0.001	0.40 (0.35–0.44)	**< 0.001**
Vasopressors	1.65 (1.48–1.84)	< 0.001	1.09 (0.95–1.24)	0.241
Mechanical ventilation	1.38 (1.24–1.53)	< 0.001	1.08 (0.94–1.23)	0.280
SOFA	1.12 (1.11–1.13)	< 0.001	0.99 (0.98–1.01)	0.509
APSIII	1.02 (1.02–1.03)	< 0.001	1.02 (1.01–1.03)	**< 0.001**
Hemoglobin	0.93 (0.91–0.95)	< 0.001	0.96 (0.94–0.99)	**0.002**
LAR	1.83 (1.75–1.90)	< 0.001	1.52 (1.44–1.60)	**< 0.001**

APSIII, acute physiology score III, CCI, Charlson comorbidity index, HR, hazard ratio, 95%CI, 95% confidence index, LAR, lactate-to-albumin ratio, SOFA, sequential organ failure assessment

**Fig. 6:**
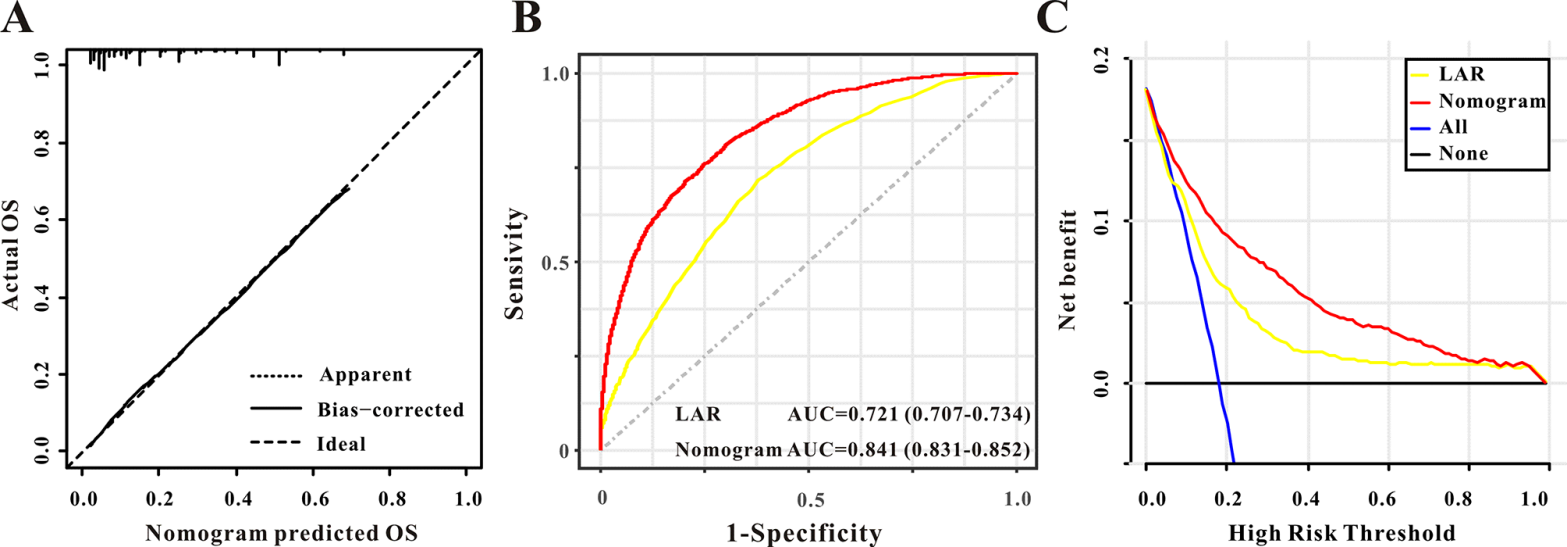
**(A)** The calibration curve for predicting in-hospital mortality. **(B)** Receiver operating characteristic curve analysis of lactate-to-albumin ratio and nomogram for in-hospital mortality prediction. **(C)** Decision curve analysis DCA of the nomogram to predict in-hospital mortality

The nomogram demonstrated a rather good model discriminative capacity for in-hospital mortality in AF patients admitted to the ICU. A significant degree of agreement was shown by the predictive nomogram’s calibration curve between the actual and predicted probabilities (Fig. 6A). In addition, the AUC for the predictive nomogram was 0.841 (0.831–0.853), exhibited excellent in-hospital mortality predictive ability (Fig. 6B). Ultimately, the clinical utility of the predictive nomogram was ascertained through the application of decision curve analysis (DCA), DCA demonstrated that the predictive nomogram was helpful for decision-making (Fig. 6C). Meanwhile, multivariate Cox regression analysis manifested that this nomogram could also independently predict in-hospital mortality when adjusted for different models, this result remains solid (Table 3).

**Table 3:** Univariate and multivariate Cox regression analysis for in-hospital mortality

Methods	HR (95%CI)	*P* value
For continuous variable		
Unadjusted	1.83 (1.75–1.90)	< 0.001
Adjusted for model I	1.88 (1.80–1.96)	< 0.001
Adjusted for model II	1.77 (1.69–1.86)	< 0.001
Adjusted for model III	1.50 (1.42–1.58)	< 0.001
For categorical variable		
Unadjusted	2.67 (2.39–2.97)	< 0.001
Adjusted for model I	2.64 (2.37–2.94)	< 0.001
Adjusted for model II	2.03 (1.82–2.27)	< 0.001
Adjusted for model III	2.01 (1.80–2.24)	< 0.001

Model I adjusted for age, gender, weight. Model II adjusted for model I plus comorbidities, drugs, interventions, score system and charlson index. Model III adjusted for model II plus laboratory results except for lactate and serum albumin. HR, hazard ratio, 95%CI, 95% confidence index, NS, not significant

## Discussion

To the best of our knowledge, this study was the first to explore the predictive role of LAR for in-hospital mortality in AF patients admitted to the ICU. Our results indicated that the optimal cut-off LAR was 0.5. Those patients with high-LAR exhibited longer length of ICU stay, length of hospital stay and higher rate of in-hospital death compared to those with low-LAR. LAR showed good in-hospital mortality predictive ability. Predictive nomogram integrated with LAR and other prognostic factors exhibited excellent discrimination and calibration abilities in predicting in-hospital mortality.

AF is the most common arrhythmia in critically ill patients in the ICU. Patients with AF make up a certain percentage of patients in the ICU, a special section where patients have high mortality risk. Hence, it’s important to explore effective intervention strategies to lower the mortality risk of AF patients in the ICU. The current top priority goal is to find simple and reliable evaluation tools for ICU physicians to distinguish high-risk AF patients in the ICU. In the present study, we aimed to use fast and routinely available variables lactate and albumin, and tried to combined lactate and albumin to predict in-hospital mortality for critically ill AF patients, which has not been reported previously. Currently, elevated LAR has been reported to be associated with poor outcomes in different cardiovascular diseases. Wang et al. reported LAR was significantly associated with 14-day, 28-day and 90-day all-cause mortality in critical patients with acute myocardial infarction [16]. Haschemi et al. [17]. used this indicator to predict survival at 30-days in patients with in-hospital cardiac arrest and found that the prognostic performance of LAR was superior to that of a single measurement of lactate or albumin for predicting survival after in-hospital cardiac arrest. In addition, Kong et al. indicated that LAR was superior to a single measurement of lactate for predicting favorable neurologic outcomes and survival to discharge after out-of-hospital cardiac arrest [18].

Our data indicated that LAR seems a good predictor for high-risk of in-hospital mortality prediction. Patients in lower LAR (< 0.5) group showed a better in-hospital survival compared to patients in higher LAR (≥ 0.5) group (HR: 2.67, 95%CI:2.39–2.97, *P* < 0.001). The present study may provide a reminder for physicians in ICU when LAR higher than 0.5, the in-hospital mortality risk significantly increased. In this study, a nomogram was plotted which incorporated with LAR, depicted excellent discrimination and calibration ability for the mortality of critically ill AF patients. Except for LAR, age, CCI, β blockers usage, APSIII score and hemoglobin were also significantly associated with in-hospital mortality in critically ill AF patients and were also finally included in the nomogram. There was no doubt that age is one of the most important predictors for prognosis prediction of AF patients [19]. Charlson comorbidity index (CCI), is a highly cited and well-established tool for predicting mortality in several clinical research. A previous study indicated that high CCI with AF had a significantly higher risk of all-cause death, stroke, major bleeding, and HF hospitalization compared to low CCI patients [20]. Our study also found higher CCI was positively associated with in-hospital mortality in critically ill AF patients. Moreover, β blockers usage was associated with significantly decrease the risk of all-cause mortality in patients with AF which verified by accumulating evidence [21]. APSIII score is a commonly-used score system for mortality prediction in ICU patients. However, usefulness of APSIII score for in-hospital mortality in critically ill AF patients has been reported previously, this study manifested that higher APSIII score is related with increased risk of in-hospital mortality. In addition, anemia is a well-recognized risk factor for adverse outcomes, the prognostic value of hemoglobin in AF patients has been proven [22, 23]. Our data also indicated that lower hemoglobin is associated with increased risk of in-hospital mortality in AF patients admitted to ICU. With the use of this nomogram, physicians could detect critically unwell AF patients at high risk of death early on and provide prompt, efficient clinical intervention to improve the patients’ prognosis.

In the present study, we found the LAR was positively associated with risk of in-hospital mortality in critically ill AF patients. The possible reasons were listed below. Since lactate is a crucial marker of blood perfusion, metabolism, and tissue oxygenation, hyperlactatemia is linked to a poor prognosis in critically ill patients, which is frequently considered as a biomarker of organ hypoperfusion in many critically ill patients. Carmona et al. reported that serum peak lactate values being reached between 4 and 24 h after cardiac surgery of AF patients, lactate was significantly predictor of major complications, mortality, and longer hospital stays [24]. Moreover, many AF patients admitted to the ICU due to fast ventricular rate associated with AF, heart failure or septic shock, acute heart failure and systemic inflammatory response lead to hemodynamic instability, thereby resulting in hyperlactatemia due to increasing plasma lactate production and decreasing clearance [25, 26]. Moreover, inflammation may contribute to the development and prognosis of AF because atrial myocytes are affected by inflammatory cell infiltration and oxidative damage [27]. As a negative acute phase reactant, albumin has an inverse relationship with oxidative stress and inflammation, the degree of hypoalbuminemia correlates with the intensity of the inflammatory response in critically ill patients [28]. Previous studies reported that low serum albumin was not only associated with new-onset AF in ICU [29] but also with mortality of AF patients [30]. Hence, high LAR value combined with hyperlactatemia and hypoalbuminemia may reflect hypoperfusion and increased systemic inflammatory response in AF patients admitted to ICU. Precise mechanisms need to be clarified in the further studies. As clinicians in ICU, if LAR is high in an AF patient, this may prompt us that this patient is in high-risk of in-hospital mortality, the clinicians should pay more attentions to check if this patient is currently in a state of hypoperfusion and infection. The clinicians may check the vital signs such as blood pressure, heart rate, oxygen saturation, etc. Furthermore, the clinicians may also focus on the cardiac function and infection status. Hence, it may effectively to lower LAR by correcting hypoperfusion through rate control or rhythm control management, heart failure improvement and anti-infection treatment and reduce the mortality in AF patients admitted to the ICU.

Several limitations should be addressed in this study. First, the present study a retrospective study based on public database, the results should be verified by further prospective study. Secondly, we only measured the baseline levels of LAR 24 h after the admission, thus dynamic monitoring of LAR while a patient is in the hospital would be more accurate in this regard. Thirdly, when evaluating the lactate/albumin ratio to estimate the prognosis of critical illness AF patients, it is important to take into account the patients’ general condition because lactate and albumin levels are influenced by a variety of circumstances. Moreover, this study was based on MIMIC-IV database, some information we need are not included in this public database, included types of AF (paroxysmal AF and persistent types of AF), mitral regurgitation presence or not, CHA2D2-VAsc score, pathology (valvular disease, sepsis, etc.), further studies are needed to take these factors into consideration.

## Conclusions

This study provided evidence that elevated LAR was positively associated with increased risk of in-hospital mortality in AF patients admitted to the ICU. The prognostic significance of the LAR in critically ill patients with AF should be investigated in more prospective trials in the future.

### Electronic supplementary material

Below is the link to the electronic supplementary material.

Supplementary Material 1

## Data Availability

The dataset used in this study, MIMIC-IV, is available at https://mimic.physionet.org/.
