# Correction: Usefulness of lactate to albumin ratio for predicting in-hospital mortality in atrial fibrillation patients admitted to the intensive care unit: a retrospective analysis from MIMIC-IV database

**DOI:** 10.1186/s12871-024-02706-3

**Published:** 2024-08-31

**Authors:** Ting Huang, Sen Lin

**Affiliations:** 1 Department of Cardiology, The Central Hospital of Wuhan, Tongji Medical College, Huazhong University of Science and Technology, Wuhan , 430014 Hubei China; 2 Key Laboratory for Molecular Diagnosis of Hubei Province, The Central Hospital of Wuhan, Tongji Medical College, Huazhong University of Science and Technology, Wuhan , 430014 China; 3 Department of Cardiology, Zhongnan Hospital of Wuhan University, Wuhan, 430071 Hubei China

**Correction: BMC Anesthesiol 24**,** 108 (2024)**

10.1186/s12871-024-02470-4

Following publication of the original article [1], the authors reported an error in affiliation 1 and a missing affiliation for author Ting Huang.

Incorrect:

Department of Cardiology, Tongji Medical College, The Central Hospital of Wuhan, Huazhong University of Science and Technology, Wuhan 430014, Hubei, China.

Correct:

Department of Cardiology, The Central Hospital of Wuhan, Tongji Medical College, Huazhong University of Science and Technology, Wuhan 430014, Hubei, China.

The missing affiliation is:

Key Laboratory for Molecular Diagnosis of Hubei Province, The Central Hospital of Wuhan, Tongji Medical College, Huazhong University of Science and Technology, Wuhan 430014, China.

The affiliations has been updated above and the original article [1] has been corrected.
